# Standardized videos in addition to the surgical curriculum in Medical Education for surgical clerkships: a cohort study

**DOI:** 10.1186/s12909-022-03314-w

**Published:** 2022-05-19

**Authors:** J. W. Selten, T. Nazari, E. H. Andriessen, S. Konings, T. Wiggers, J. de Jonge

**Affiliations:** 1 Department of Surgery, Erasmus University Medical Center, Doctor Molewaterplein 40, 3015 GD Rotterdam, the Netherlands; 2 Incision Academy, Amsterdam, the Netherlands

**Keywords:** Medical education, Surgery, Clerkships, Video

## Abstract

**Background:**

Medical students are expected to translate the theoretical knowledge gained during their study to practical knowledge during the clerkships. A surgical educational platform with standardized videos may be the solution. However, the effects of a structured online video-based platform in addition to the standard curriculum on students’ self-reported and tested surgical knowledge during the surgical clerkship must be assessed.

**Methods:**

Fourth-year medical students (*n* = 178) participated in a 6-week course of theoretical and practical training followed by a 10-week in-hospital clerkship in the Erasmus University Medical Center (Erasmus MC), Rotterdam, The Netherlands and 11 affiliated general hospitals. Ninety students followed the usual surgical curriculum (control group), followed by 88 students who were given voluntary access to a video-based surgical educational platform of Incision Academy (video group). At the start (T0) and end (T1) of the clerkship, both groups filled out a surgical knowledge test and a survey regarding their self-reported surgical knowledge and their access to available study sources. Supervisors were blinded and surveyed concerning students’ performance and their acquired knowledge. We analyzed the data using paired and unpaired student t-tests and linear regression.

**Results:**

At the end of the clerkship, students in the video group indicated that they had better resources at their disposal than the control group for surgical procedures (*p* = 0.001). Furthermore, students in the video group showed a greater increase in self-reported surgical knowledge during their clerkship (*p* = 0.03) and in more objectively tested surgical knowledge (*p* < 0.001).

**Conclusions:**

An online surgical educational platform with standardized videos is a valuable addition to the current surgical curriculum according to students and their supervisors. It improves their test scores and self-reported surgical knowledge. Students feel better prepared and more able to find the information necessary to complete the clerkship.

**Trial registration:**

Registry not necessary according to ICMJE guidelines.

**Supplementary Information:**

The online version contains supplementary material available at 10.1186/s12909-022-03314-w.

## Introduction

The understanding of surgical anatomy and procedures is important for medical students, regardless of their future specialty. However, the first 2 to 4 years of medical school are comprised predominately of theoretical learning and rarely include clinical experience in the operation room [1]. This makes the transition to the clinical part of their education challenging. An earlier study has shown that instruction in performing specific skills and procedures improves individuals’ evaluated competence in performing that skill [2].

In the Netherlands, the medical training consists of a three-year bachelor program and a three-year master program, but the specific content of the curricula may vary among universities [1]. The bachelor program is predominantly theoretical learning and the master program involves practical learning through clerkships. The surgical master program at the Erasmus University Medical Center (Erasmus MC), Rotterdam, The Netherlands consists of a 6-week preparatory course prior to a 10-week surgical clerkship. The preparatory course involves e-learnings, web-lectures, and texts from books and articles, accompanied by hands-on workshops, discussion meetings, and lectures led by attending physicians or faculty.

Although medical students complete a 6-week preparatory course together, for the 10-week clerkship students are allocated in the academic or affiliated general hospitals. These hospitals all have their specialties and differences in patient populations based on their unique clinical profile. Students, therefore, might not have the opportunity to see the same surgical procedures. It may thus occur that some medical students see liver transplantations and Whipple’s procedures whereas other see general surgical procedures such as inguinal hernia repair and laparoscopic cholecystectomies. Additionally, individual surgeons might have their own approach and preferences for a certain procedure which hinders students to learn a standardized approach. Lastly, not all surgeons provide the same explanation before, during, or after surgical procedures and not all students dare to ask questions about the procedures in the operating room (OR). However, a similar level of understanding of basic surgical procedures for all students at the end of their medical training is important, regardless of their future specialization. To cope with this unbalance in the clinical palette between different clinics and with the instruction styles of individual surgeons, a list of 10 common practical key surgical procedures was composed that all our students should have knowledge about at the end of their clerkship (Table 1).

**Table 1:** Ten preselected courses that were considered mandatory for all students (control group and video group) to study during the clerkship

**Procedures**			
Toenail avulsion			
Inguinal Hernia Repair - Open - Indirect Hernia			
Cholecystectomy - Laparoscopic			
Appendectomy - Laparoscopic			
Lumpectomy - General Principles			
Carpal Tunnel Release - Cadaver			
Lipoma Excision			
Abdominal Wall Incision - Midline			
Colectomy - Laparoscopic (Right)			
Colectomy - Open (Left)			

Video instruction can help to fill the gap between theoretical instruction of surgical, clinical and anatomical knowledge and expert hands-on instruction or as an independent teaching module [3]. Medical students and surgical trainees frequently use online video resources for their learning, which often includes viewing surgical videos on YouTube [3]. However, YouTube is not an accredited medical educational resource, and any individual or organization can upload videos to the site. The content is not organized by quality, but rather search results appear in order of popularity and other algorithms. Another study found that the highest-ranked laparoscopic cholecystectomy videos on YouTube displayed suboptimal technique; furthermore, half of the videos exhibited unsafe maneuvers and only 10% demonstrated a satisfactory critical view of safety [4]. Additionally, the videos lacked an explanation of pre- and postoperative aspects such as indications, complications, surgical anatomy and information regarding patient selection that were deemed essential knowledge for surgical trainees [5].

Consequently, we provided students access to a structured online video-based surgical educational platform with standardized, high-quality educational videos [6]. It has been designed to prepare students and residents for specific surgical procedures and contained over 300 surgical video demonstrations at the moment of the study, including the aforementioned 10 determined key-procedures. All available courses on the platform include a step-by-step description and video-demonstration of a skill or surgical procedure. Additionally, each course is accompanied by supplemental introductory, preoperative and postoperative textual sections, anatomical illustrations, multiple-choice examinations and in most courses also interactive anatomical 3D models.

We hypothesized that these educational videos would help to standardize and improve the learning experience of the medical students in the video group compared to students who did not have access to the platform. We also hypothesized that these videos would help to ensure that all medical students get optimal exposure to the 10 pre-defined key-procedures, regardless of the actual attendance in the different hospitals.

The purpose of this study was to assess the educational value of instructional videos on surgical knowledge of essential basic surgical procedures as well as feelings of preparedness before and during the general surgery clerkship as reported by students and their supervisors.

## Methods

### Study population

Between February 2019 and June 2019, 178 fourth-year medical students of the Erasmus MC were approached at the start of their surgical clerkships for inclusion in this study. Written consent was obtained. The study protocol was approved by the medical ethical committee of the Erasmus MC, Rotterdam, The Netherlands (METC-2019.0564).

All students participated in a 6-week preparatory course followed by a 10-week clinical clerkship. During this clerkship, students were distributed over 12 teaching hospitals in the region of Rotterdam (3-15 students per hospital, depending on the size of the hospital).

**Fig. 1:**
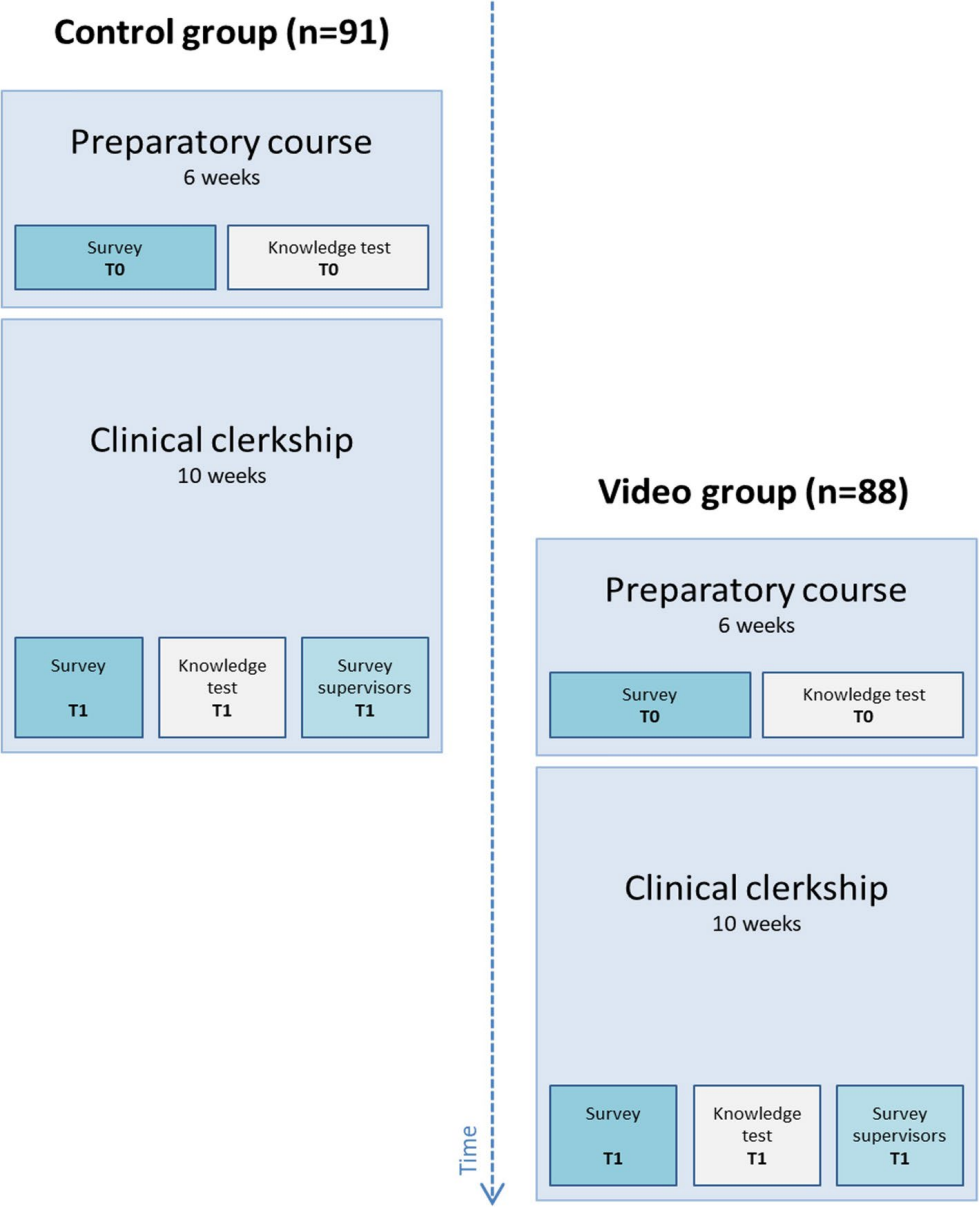
Flow-chart study design

Four hundred fifty students participate in the course and clerkship each year, divided by the university into 5 consecutive starting cohorts of 90 students on average based on progress in the first 3 years of medical school (bachelors program). For this study, we chose to use the university’s consecutive cohorts to divide the students into the “control group” and the “video group”. The first 90 students that followed the standard surgical curriculum were included in this study as the control group (Fig. 1) and were advised to study the 10 key-procedures (Table 1). The second group of 90 students was included as the video group and also followed the standard surgical curriculum, and in addition received unlimited, voluntary access to a structured online video-based surgical educational platform on surgical procedures, skills, and anatomy [6]. Students in the video group had access to all courses including the videos on the online platform and were advised to complete the ten preselected courses covering the key-procedures (Table 1). These 10 cases were chosen based on the incidence in general and abdominal surgery.

### Surveys

Both the control and video group were given a survey at the end of their 6-week preparatory course (T0) and at the end of their 10-week clerkship (T1), which they could fill in anonymously (Fig. 1). The first survey (T0) included questions related to the student demographics and preparation for the clerkship (Additional file 1: Appendix 1). Questions were about two domains: 9 questions regarding self-reported knowledge on surgical anatomy, surgical objectives, and complications. Overall student self-assessment was rated on a 5-point Likert scale. This survey was followed by a knowledge test including questions focused on the surgical and anatomical knowledge of the students (Additional file 2: Appendix 2). The questions concerning students’ knowledge included different surgical disciplines (general, oncological, orthopedic/trauma, and gastrointestinal) and did not influence the students’ official grades. The questions were written by 4 teachers involved in this study (J.S., E.A, T.W., J.J.) whom all have experience in writing test questions for medical student examinations and educational surveys. Students were not informed about the results of the pretest after they completed it. The second questionnaire (T1) included 35 questions concerning their experiences (observed surgical procedures and preparation time) during the clerkship, the multimedia sources they used in preparation of and during the clerkship, and their overall opinion regarding these 10 weeks (Additional file 3: Appendix 3 (control group) and Additional file 4: Appendix 4 (video group)). The knowledge test at T1 covered the same topics as in the pre-test (Additional file 5: Appendix 5). All questionnaires (T0 and T1) in both groups were filled in on paper in the presence of an attending physician to prevent students from looking up the correct answers and discussion amongst students. Data about user activity on the Incision Academy were directly retrieved from the platform.

At the end of the clerkship (T1), all supervising surgeons in affiliated hospitals received a digital questionnaire regarding the surgical knowledge and overall performance of the medical students on a 5-point Likert scale (Additional file 6: Appendix 6). This questionnaire was not about individual students, but the groups as a whole and was, therefore, sent twice – once for the control group, and once for the video group (Fig. 1). Supervising surgeons were informed that a study was being conducted on the availability of study sources for students during the clerkship, they were however blinded and not informed that the video group had access to an online video-based surgical educational platform.

### Outcomes and definitions

The difference in self-assessed knowledge and test results between the control group and the video group was the primary outcome of the study. Secondary outcomes were the length of preparation time, number of observed surgical procedures, types of study sources students used to study, and their evaluation. Also, the overall evaluation of both groups on group level by the supervising surgeons was considered a secondary outcome.

### Statistical analysis

The Shapiro-Wilk test was used to test normality. Normally distributed data were analyzed using paired Student t-test when comparing pre- and post-procedural knowledge test results. The unpaired Student t-test was used when comparing results based on differences in demographics, course participation, and post-clerkship (T1) results between groups. The non-normal distributed data were described by the median and range or interquartile range (IQR). The Mann-Whitney *U* test was used to compare ordinal data between groups and the χ^2^ test was used for categorical data. Differences in outcome measures between T0 and T1 in both groups were compared by using linear regression.

All statistical analyses were done with SPSS (IBM Corp. Released 2016. IBM SPSS Statistics for Windows, Version 24.0. Armonk, NY: IBM Corp.). A *p*-value of less than 0.05 was considered statistically significant.

## Results

### Baseline characteristics

A total of 91 students were included in the control group and 86 in the video group. In the control group 60 students (66%) filled in the survey at T0 and 46 (51%) at T1. In the video group, 77 students filled in the survey at T0 (90%) and 45 (52%) at T1.

Participants in the control and video groups did not differ in age (mean 24.5, SD 2.7 years vs mean 24.7, SD 3.3 years; *p* = 0.85) or gender (39 female (65%) vs 54 female (70%); *p* = 0.39). Students also did not differ in prior surgical experience (*p* = 0.42) nor in their desire to pursue a career in surgery at T1 (*p* = 0.84) (Table 2).

**Table 2:** Comparison of baseline characteristics between the control group and the video group

	**Control group (n=60)**	**Video group (n=77)**	*p value*
Gender			0.39
Female (%)	39 (65)	54 (70)	
Male (%)	21 (35)	23 (33)	
Mean age (SD)	24.5 (2.7)	24.7 (3.3)	0.85
Experience†			0.42
No prior experience	28	28	
Work experience in non- surgical specialty	23	34	
Work experience in surgical specialty	9	12	
Pursue career in surgery‡	3 (2-4)	3 (2-4)	0.84

† Practical working experiences (e.g. student job) in a hospital before the current internship

‡ I would like to pursue a career in a surgical specialty (1 = definitely not - 5 = yes, definitely)

### Self-reported surgical knowledge and test results

**Fig. 2:**
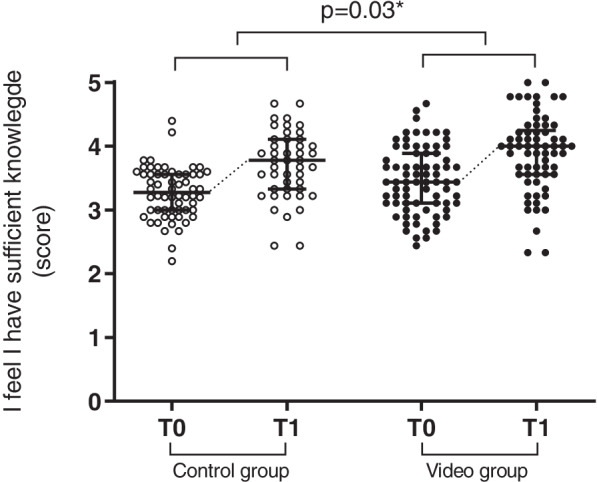
Responses to survey questions regarding self-reported knowledge combined of students in the control group (clear dots) on T0 and T1 and students in the video groups (black dots) on T0 and T1 (median + IQR). The increase in score over time was significantly greater in the video group than in the control group (*p* = 0.03)

**Fig. 3:**
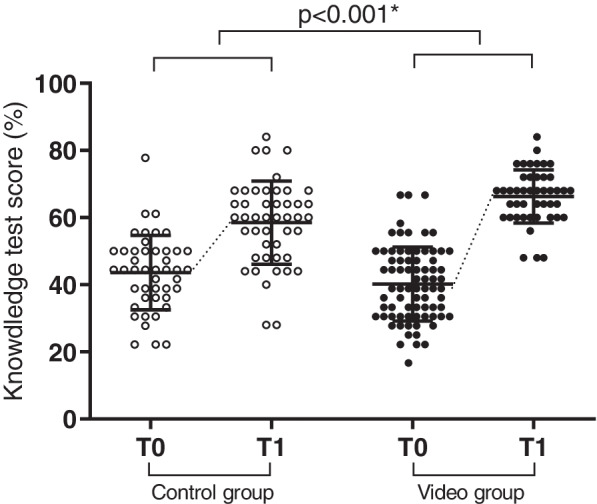
Percentage of questions correctly answered in the control group (clear dots) on T0 and T1 and correctly answered questions in the video group (black dots) on T0 and T1. Both groups performed better at the end of the clerkship (T1) than at the start (T0) (mean + SD). The increase in the video group was significantly greater than in the control group (*p* < 0.001*) as indicated by the dotted line

The internal consistency of the measurements at T0 and T1 were reliable. Students in the control group had a marginally better self-reported knowledge at the start of the clerkship (Table 3). However, the increase in self-reported knowledge during the clinical clerkship in the video group was significantly greater (*p* = 0.03 Fig. 2). Furthermore, students in the control group scored slightly better on tested surgical knowledge prior to the clerkship (Table 3). Similarly, the increase in the percentage of correctly answered questions was significantly greater in the video group. Scores on the knowledge test in the control group increased from 43.6 (SD 10.9) to 58.5 (SD 12.4) versus 40.2 (SD 11.0) to 66.3 (SD 7.9) in the video group (*p* < 0.001, Fig. 3).

**Table 3:** Results survey at T0 andT1 for both groups

	Control group		Video group		*p*-value
	Before start (T0)	End (T1)	Before start (T0)	End (T1)	
**I feel I have sufficient sources**					
Cronbach’s α	0.72	0.88	0.76	0.84	0.001
Median (IQR)	3.6 (3.2 – 4.0)	3.8 (3.3 – 4.2)	3.5 (3.0 – 4.0)	4.2 (3.8 – 4.6)	
**I feel I have sufficient knowledge**					
Cronbach’s α	0.69	0.77	0.77	0.81	0.03
Median (IQR)	3.4 (3.0 – 3.7)	4.0 (3.7 – 4.2)	3.2 (3.0 – 3.7)	4.1 (3.9 – 4.4)	
**Multimedia**					
Cronbach’s α		0.80		0.80	0.002
Median (IQR)		3.9 (3.6 – 4.3)		4.4 (3.8 – 4.8)	
**Knowledge test scores (%)**					
Mean (SD)	43.6 (10.9)	58.5 (12.4)	40.2 (11.0)	66.3 (7.9)	0.0001

### Observed surgical procedures

The median number of self-reported observed surgical procedures did not vary significantly between groups (*p* = 0.83); median 50 (IQR 40 – 66) in the control group and median 50 (IQR 40 – 80) in the video group. Neither did the self-reported number of surgical procedures in which the student was able to participate actively or was scrubbed in; median 30 (IQR 22 – 40) in the control group and median 30 (IQR 20 – 46) in the video group (*p* = 0.836).

Furthermore, the self-reported time spent on preparation for a procedure did not differ between the control and video group; median 30 min (IQR 20 – 45 min.) in the control group and median 30 min. (IQR 19 - 45 min.) in the video group (*p* = 0.17).

### Study sources

On average at T1, students reported using 4 different sources to prepare themselves for procedures (median 4 (IQR 3 – 5) in the control group and 4 (IQR 3 – 4) in the video group, *p* = 0.79). In both groups, a Dutch website dedicated to operative notes and descriptions was most frequently reported as an important source (65% in the control group and 62% in the video group). Furthermore, in both groups, almost half of the students reported using YouTube [7] as one of their main sources for preparing for surgical procedures (*n* = 24 (52%) and *n* = 22 (49%); *p* = 0.76). Other sources included other internet sites (e.g. UpToDate [8], MedInfo [9]; 29% overall), anatomy books (e.g. Gray’s Anatomy [10], Sobotta [11]; 78% overall), guideline databases (e.g. AO trauma [12], Guidelines by the Dutch Federation of Medical Specialists [13]; 27% overall) and the e-learnings and lectures from the course prior to the clerkship (31% overall).

The earlier described Incision Academy video platform had 2221 visits by 79 students (92%) (mean 27 visits per student) in the 10 weeks the website was available to them. Only 3 students with an account did not use their account (4%). At the time of this study, the website hosted 374 courses and students watched a total of 257 different courses (69%) and completed 136 of these courses (53%).Of the 10 recommended courses, the “laparoscopic cholecystectomy” was viewed most often (*n* = 51) but the “partial toenail avulsion” course was viewed the least number of times (*n* = 19). Outside of these recommended courses, students most often viewed the “Totally Extraperitoneal (TEP) Repair”(*n* = 36). There was a clear peak in use of the platform during the clerkship, more specifically on weekdays. No videos were viewed during the preparatory course prior to the clerkship (data not shown).

**Fig. 4:**
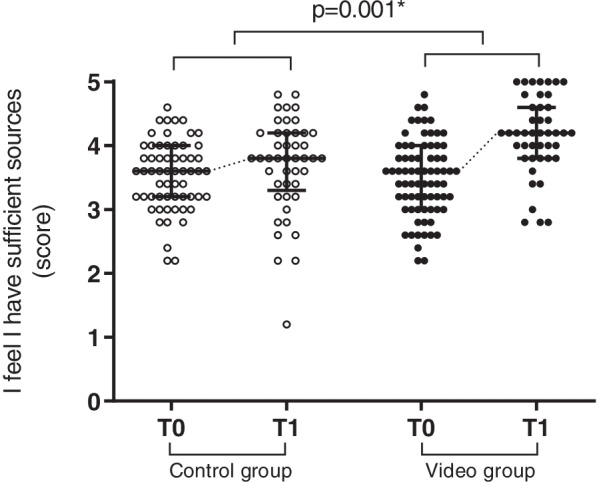
Responses to survey questions regarding the availability of study sources combined of students in the control group (clear dots) on T0 and T1 and students in the video groups (grey dots) on T0 and T1 (median + IQR). Scores in the video group increased significantly more than those in the control group (*p* = 0.001) as is represented by the dotted line between medians

**Fig. 5:**
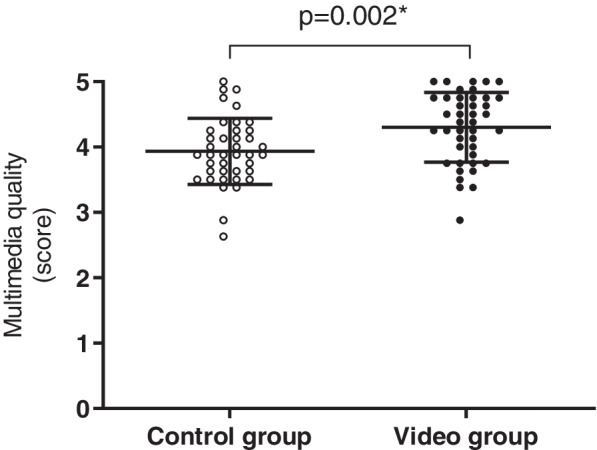
Responses to survey questions regarding multimedia quality and usefulness combined in one factor of students in the control group (clear dots) and the video group (black dots) on T1 (median + IQR). Students in the video group rated the quality of the multimedia they used significantly higher than those in the control group (*p* = 0.002)

Students in both groups started their clerkship with a low overall rating of the availability of study sources to prepare themselves for the clerkship, general procedures, basic surgical skills, specialized surgical procedures, and surgical anatomy (Fig. 4). At the end of the clerkship, both groups showed an increase in the evaluation of the availability of study sources, although this increase was significantly greater in the video group (control group: ∆0.2; video group: ∆0.7, *p* < 0.001). Furthermore, the students in the video group rated the quality of the multimedia sources they used significantly higher (control group; 3.9 (IQR 3.6 – 4.3), video group; median 4.4 (IQR 3.8 – 4.8), Table 3; Fig. 5). The vast majority (98% of students) rated the online video platform as indispensable for future students in the clerkship.

### Supervising surgeons

Of the 12 participating hospitals, 10 supervising surgeons filled in the survey concerning students in the control group (83%) and 7 for the video group (58%). They had a median of 5 years of experience in supervising medical students (range 1 – 20 years) and all of them had input from their colleagues on all of the students when giving feedback and filling out the survey. On questions regarding students’ knowledge (Cronbach’s α = 0.798), the control group scored lower than the video group (median 2.9 (IQR 2.7 – 3.5) versus 3.5 (IQR 3.3 – 4.0)); which was not significantly different due to the small number of participants (*p* = 0.17). On questions regarding the availability of study sources (Cronbach’s α = 0.837), the video group scored higher (median 3.75 (IQR – 3 – 4.3)) than the control group (median 3.4 (IQR 3 – 3.8)), *p* = 0.49). The self-confidence of students in general and in the OR specifically (combined factor Cronbach’s α = 0.856), was similar (median 3 versus 3). However, 3 out of 10 supervisors ranked the self-confidence of students “low” in the control group, versus none in the video group.

Six of the 10 surgeons indicated students in the control group lacked high-quality video material to prepare themselves for procedures. However, no supervisor indicated students lacked preparation material in the video group, and 2 of them specified that they noticed significant improvements in peri-operative knowledge in students in this group.

## Discussion

Students are expected to acquire surgical skills and knowledge during the clinical clerkships, while time and exposure to different procedures are limited. The need to ensure similar medical education to all students, in a landscape of increasing differentiation among surgical (sub) disciplines and hospitals and thus decreasing exposure to all facets of surgery, is a challenge to every medical school. However, newer learning tools, such as online surgical videos and courses to supplement traditional didactic lectures and hospital-based learning are becoming available. In this study, we found that surgical knowledge increased significantly during the surgical clerkship when students are provided access to a structured online video-based surgical education platform in addition to the standard surgical curriculum. More importantly, surgical knowledge was more uniformly spread in students in the video group, despite the inhomogeneous exposure to different “live” surgical procedures due to differences in teaching hospitals. Also, self-reported knowledge increased significantly over the course of the clerkship in the video group compared to students who did not have access to this platform and who used publicly available sources. It is interesting to note that the control group had previously out-performed their peers in the test group, but this advantage was seemingly negated by the use of the video platform. Students felt more prepared due to better tools and sources for the clerkship in general and for the different interventions they were going to see specifically. Because the access to the video’s did not stop for all student immediately after the surgical clerkship, several students also viewed video’s for the urology and gynecology courses (*n* = 62). This shows that students had a positive experience with the platform and continued to use it beyond the scope of our study. They almost unanimously rated the platform essential for future students in the clerkship. Furthermore, the supervising surgeons (*n* = 10) in this study rated the knowledge of the students in the video group higher compared to the control group.

Earlier studies have reported comparable positive results of increased knowledge and self-confidence of students from educational surgical videos [14–17]. Several features of this type of education can explain these effects. First, students have the time to prepare themselves beforehand by viewing an uncomplicated procedure, and therefore know what to expect. These videos can be watched and -selectively - re-watched at the students’ desired pace and moment (just in time). Consequently, they will be able to concentrate better on the next procedural steps during the live operation and have fewer problems determining surgical anatomy. Feeling prepared also increases student confidence and therefore facilitates optimal learning in the operating theatre [18–20].

Furthermore, the structured step-by-step explanations of surgical procedures decrease the cognitive load by fragmentation of the study material [16]. The structured pre-, per- and post-operative objectives form an essential part of the preparation for the procedure and provide relevant information based on validated sources and guidelines. This is especially important because inexperienced students may not be able to tell if a shown procedure on a non-official platform is following national or international guidelines [21, 22]. Although the majority of students use YouTube as a learning tool in medical school, recent studies have shown that half of the educational videos of laparoscopic cholecystectomies on YouTube showed dangerous situations and only 10 followed the international guidelines in demonstrating the “critical view of safety” [4, 23, 24]. Another review that focused on videos on the treatment of distal radius fractures found that only 16 of the 68.000 videos met the international criteria [25]. Videos of knee arthrocentesis were deemed suitable for educational purposes in 62% of cases [21]. A study focusing on face-lift procedures points out that videos for educational purposes did not cover pre- and postoperative aspects as indications, complications, and patient selection [26]. These results indicate that students need to be cautious when using YouTube videos in their learning and preparation [3, 27–31]. The courses on the online video-based surgical education platform in this study follow international guidelines and are supervised by expert surgeons, anatomists, and surgical educators.

In this study, the use of this platform by students was voluntary. By incorporating the platform as an obligatory learning and teaching tool for students and teachers, the learning yield may increase. And although this study or its contents were not officially included in the final exams, we did see overall higher scores on the exam in the video group (data not shown). Additionally, students used the platform solely during the clinical clerkship and not during the preparatory course. This might be explained by the extensive number of texts, videos and cases they have to study during the course prior to the clerkship. However, this also shows that students indeed use the videos as a tool to prepare themselves for the procedures they are going to encounter in the hospitals.

Lastly, structured, high quality, educational videos offer a more homogeneous education for students independent of the surgeons and procedures they encounter during their clerkship. One of the problems we wanted to overcome with the incorporation of an online video-based educational platform was the differences in surgical exposure for the students enrolled in the academic center (Erasmus MC) or one of the eleven affiliated community hospitals. Even though several studies have found no differences in performance and study results between students in academic or community hospital clerkships, we found in this study that students assigned in the university center for the clerkship reported longer preparation times for procedures (data not shown) [32, 33]. Procedures performed in tertiary centers are generally more complicated and information on these procedures is less readily available and more complex. Understandably, these students also reported lower numbers of observed interventions and fewer operations in which they actively participated. However, the location of the clerkship did not affect students’ test scores on the knowledge test (*p* = 0.06) or the mandatory university test (*p* = 0.15; data not shown).

### Strengths and limitations

Unfortunately, randomization in this study was unfeasible. Due to the nature of the intervention in the video group, two consecutive cohorts were needed to avoid contamination of the groups by cross-contact of the students in daily life or via social media as much as possible. As mentioned, the selection for these consecutive cohorts was made by the university and based on students’ progress in the previous 3 years of medical school. Therefore, students that progressed faster through the first 3 years were placed in the first group (control group), and the students that required more time for the first 3 years in the second group (video group). This effect is visible in the knowledge test scores at T0 where the control group had slightly higher scores (*p* = 0.09), but that is reversed at T1 with higher scores for the video group, underlining the effect of the intervention (*p* = 0.001).

Although a large number of students participated in this study, the decreasing number of students filling out the second survey might overestimate the increase in self-reported knowledge and test scores. Highly motivated students may engage more in available study materials and may have been more eager to fill out the surveys. We did see a difference between the control and video group in the number of surveys filled out at T0 (66% vs 90%) because the second group had already had an introduction to the platform prior to filling out the first survey. This difference in the number of filled out surveys was not seen at T1. Furthermore, we did not see a significant difference in students interested in a career in a surgical specialty in both groups or in scores on the knowledge test in students that indicated to be interested in a career in surgery and those who were not.

Also, because theoretical knowledge is the easiest to test, this might misjudge technical abilities, clinical thinking, and skills like communication, professionalism, and teamwork in students. Although we did see a clear trend in more positive opinions of the surgeons on students in the video group in this regard, these results were not significantly different due to the small number of surgeons in our cohort.

### Implications

This study demonstrates intensive use by students, an increase in self-reported and tested knowledge, and better evaluations of supervising surgeons. This is especially true in the case of differences in exposure that occurs between hospitals. With increased use, the database of courses including videos can be expanded to include more complex operations and different approaches to certain procedures. When used internationally, a more standardized universal language for surgical procedures can be created which may facilitate (research) collaborations in the future within the surgical community and beyond.

## Conclusion

The addition of high quality and structured video courses of surgical procedures and skills to standard surgical curriculum improved self-reported knowledge and tested knowledge in students during their surgical clerkship. Student satisfaction regarding the availability of high-quality study sources was higher in the video group and students felt more prepared for the clerkship in general and for specific procedures. Furthermore, supervising surgeons scored the knowledge and skills of the students with access to the online video platform higher. The various courses on this platform facilitate learning objectives prior to the procedure students will see or participate in and ensure a homogeneous surgical experience for all students. Based on the findings in our study, we suggest providing access to a high-quality video platform to all students in the clinical phase of their training.

## Supplementary Information

**Additional file 1. **Student questionnaire - T0 - Demographics.

**Additional file 2. **Student questionnaire - T0 - Surgical knowledge.

**Additional file 3. **Student questionnaire - T1 - Demographics - control group.

**Additional file 4. **Student questionnaire - T1 - Demographics - video group.

**Additional file 5. **Student questionnaire - T1 - Surgical knowledge.

**Additional file 6. **Supervisor questionnaire - T1.

## Data Availability

The datasets used and analyzed during the current study are available from the corresponding author on reasonable request. The datasets might be used for further research on the subject and is therefor not publicly available at the time.

## Abbreviations

IQR: Interquartile Range
METC: Medical ethical committee
OR: Operating Room
SD: Standard deviation
T0: Time at the start of the clerkship
T1: Time at the end of the clerkship
