# Supplementary material for: Standardized videos in addition to the surgical curriculum in Medical Education for surgical clerkships: a cohort study
Source: BMC Med Educ. 2022 May 19;22:384. doi: 10.1186/s12909-022-03314-w (PMC9121575; doi:10.1186/s12909-022-03314-w)

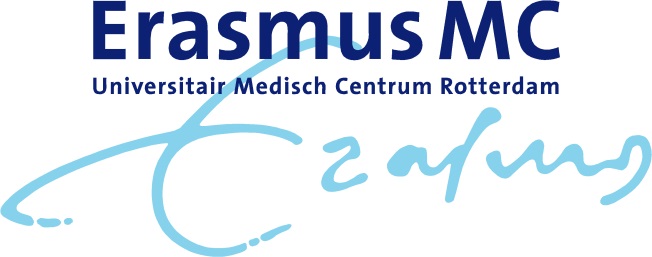


**EMC department of surgery: Surgical Internship and Multimedia**

This questionnaire is made by the department of Surgery to study the use of multimedia of interns during the clinical part of the internship.

Dear student,

The department of Education of Surgery in the Erasmus Medical Center is currently conducting a study on the use of multimedia (books, internet sources etc.) during the internship. The results will be used to improve the current curriculum. Therefore, we would kindly like to ask you to fill out the following forms (20 min. approximately). There will be 3 gift cards awarded via lottery (€20,- bol.com).

Please note that the information from these questionnaires will **not** be used in the final grading of the internship or and does not have any other implications. Your contact information will only be used to contact you if you won one of the gift cards.

By signing this form, I agree to the use of my information by the department of surgery.

| Name |  |
| --- | --- |
| Student number |  |
| E-mail |  |
| Date |  |
| Signature |  |

**Preparation**

1. Practical working experiences (student job) in hospital before current internship

- Skillslab (anatomy)
- Operating rooms (e.g. perfusion team)
- Nutrition team (surgical ward)
- Acute care student team
- “Zorgacademie”
- Education Service Center (onderwijs service centrum)
- No practical experience
- Experience in another non-surgical department
- Other:

1. I would like to pursue a career in a surgical specialty


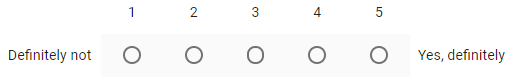


**Available sources**

Books, reliable internet sources etc.

1. I feel I have sufficient sources to prepare myself for the surgical internship(s)


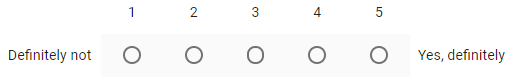


1. I feel I have sufficient sources to prepare myself for basic surgical skills


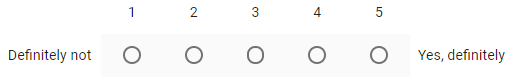


1. I feel I have sufficient sources to prepare myself for general procedures


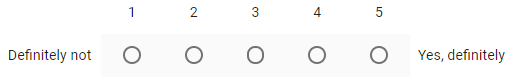


1. I feel I have sufficient sources to prepare myself for surgical procedures


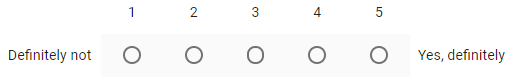


1. I feel I have sufficient sources to study surgical anatomy


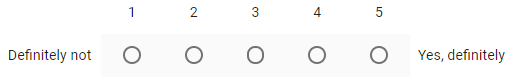


**Surgical knowledge**

1. I feel I have sufficient knowledge to prepare myself for the surgical internship(s)


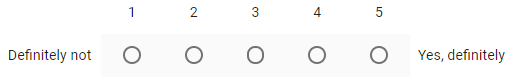


1. I feel I have sufficient knowledge about basic surgical skills

For example: knots, sutures, instruments


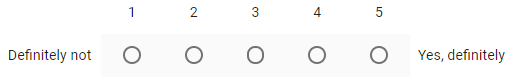


1. I feel I have sufficient knowledge of general procedures

Catheter placement, I.V., etc.


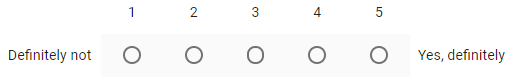


1. I feel I have sufficient knowledge about surgical procedures


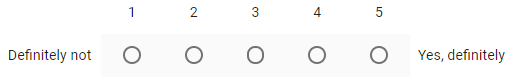


1. I feel I have sufficient knowledge about operations and surgical procedures in general


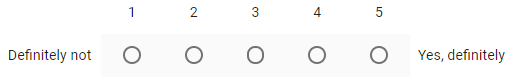


1. I feel I have sufficient knowledge about complications after surgery


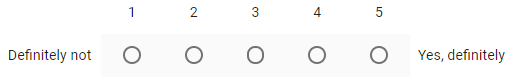


1. I feel I have sufficient knowledge about the objectives of surgical procedures


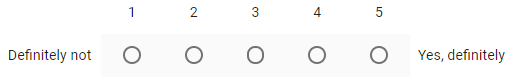


1. I feel I have sufficient knowledge about the do's and don'ts on the O.R.


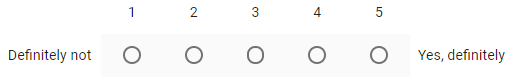


1. I feel I have sufficient surgical anatomical knowledge


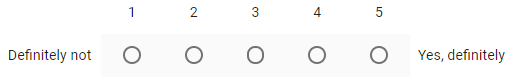

Supplement: Supplementary file 1 — Additional file 1. Student questionnaire - T0 - Demographics. [file 12909_2022_3314_MOESM1_ESM.docx]
