# Supplementary material for: Standardized videos in addition to the surgical curriculum in Medical Education for surgical clerkships: a cohort study
Source: BMC Med Educ. 2022 May 19;22:384. doi: 10.1186/s12909-022-03314-w (PMC9121575; doi:10.1186/s12909-022-03314-w)
Supplement: Supplementary file 2 — Additional file 2. Student questionnaire - T0 - Surgical knowledge. [file 12909_2022_3314_MOESM2_ESM.docx]

**Appendix 2 - Surgical knowledge – T0**

1. **What is the most commonly used approach to perform a cholecystectomy?**
2. Laparoscopic cholecystectomy
3. Open retrograde cholecystectomy
4. Open antegrade cholecystectomy
5. Transvaginal Natural Orifice Endoscopic Cholecystectomy
6. Robot-assisted laparoscopic cholecystectomy
7. **Which two structures should be transected after dissecting the peritoneal envelope during a laparoscopic cholecystectomy?**
8. Cystic duct and cystic artery
9. Common bile duct and cystic artery
10. Cystic duct and right hepatic artery
11. Common hepatic duct and right hepatic artery
12. Common bile duct and right hepatic artery
13. **In which order should the following steps be performed when performing a right hemicolectomy?**

I. Transection of the hepatocolic ligament
II. Opening the lesser sac
III. Incising the line of Toldt

Answer: _________________________

1. **In what order is the dissection of the appendix performed during a laparoscopic appendectomy?**

A. adhesions - appendicular base - meso-appendix

B. meso-appendix - appendicular base - adhesions

C. adhesions - meso-appendix – appendicular base

D. appendicular base - adhesions - meso-appendix

E. appendicular base - meso-appendix - adhesions

1. **What is the initial treatment of an appendicitis in the occurrence of a large appendicular abscess?**

A. Open appendectomy

B. Percutaneous drainage

C. Laparoscopic appendectomy

D. Wait and see policy

E. Intravenous antibiotics

1. **Which numbers in *Figure 1* point out the head of the pancreas and the gonadal artery, respectively?** *Please circle the correct numbers*.


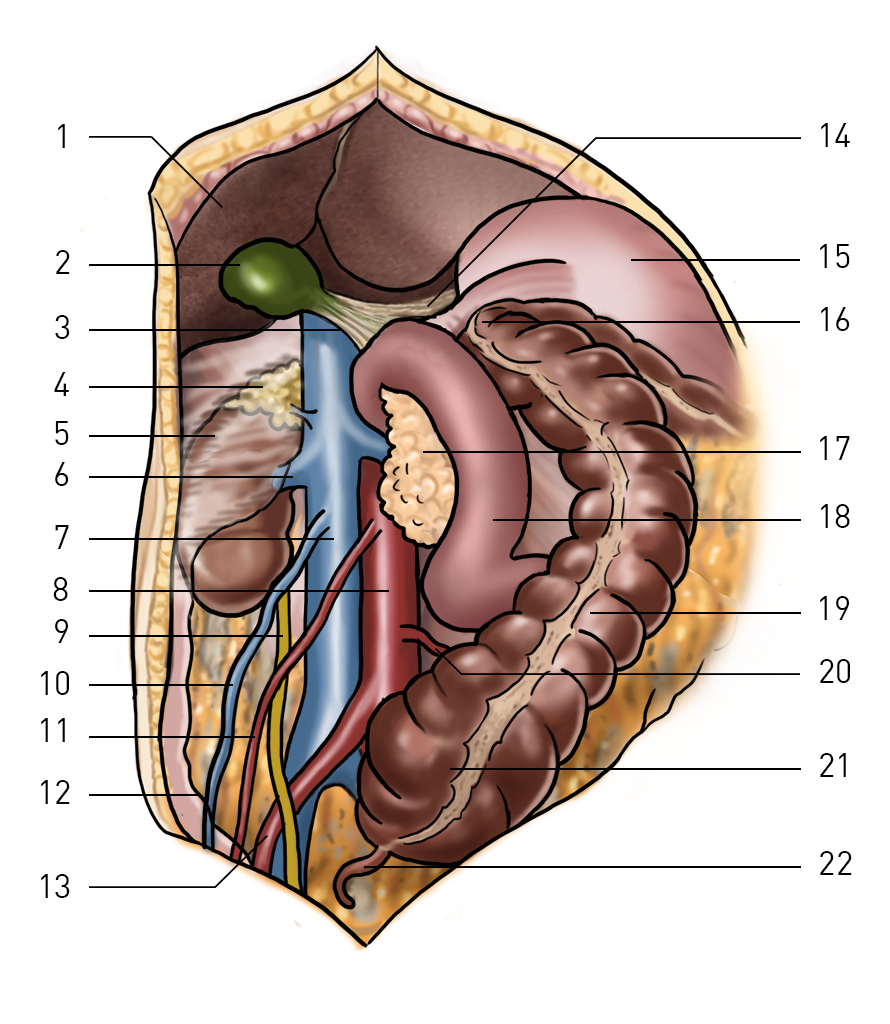


*Figure 1*


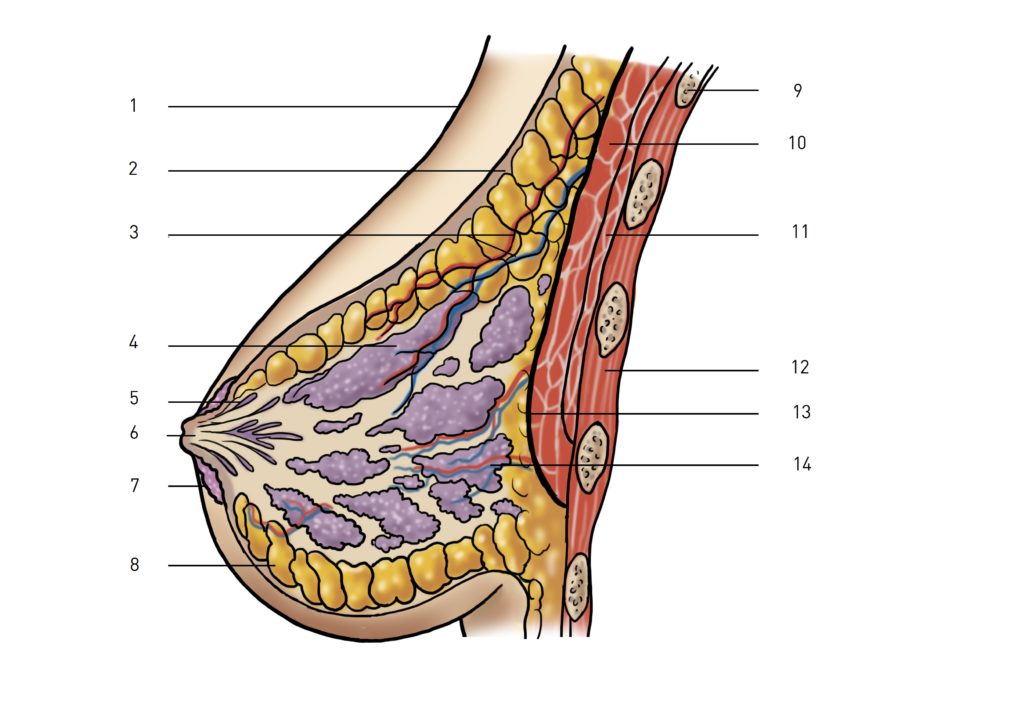


*Figure 2*

1. **Which structure is indicated by number 5 in *Figure 2*?**
2. Mammary vessels
3. Lobes of the mammary gland
4. Lactiferous duct
5. Fatty tissue
6. Nipple
7. **What structure is indicated by number 3 in *Figure 3*?**


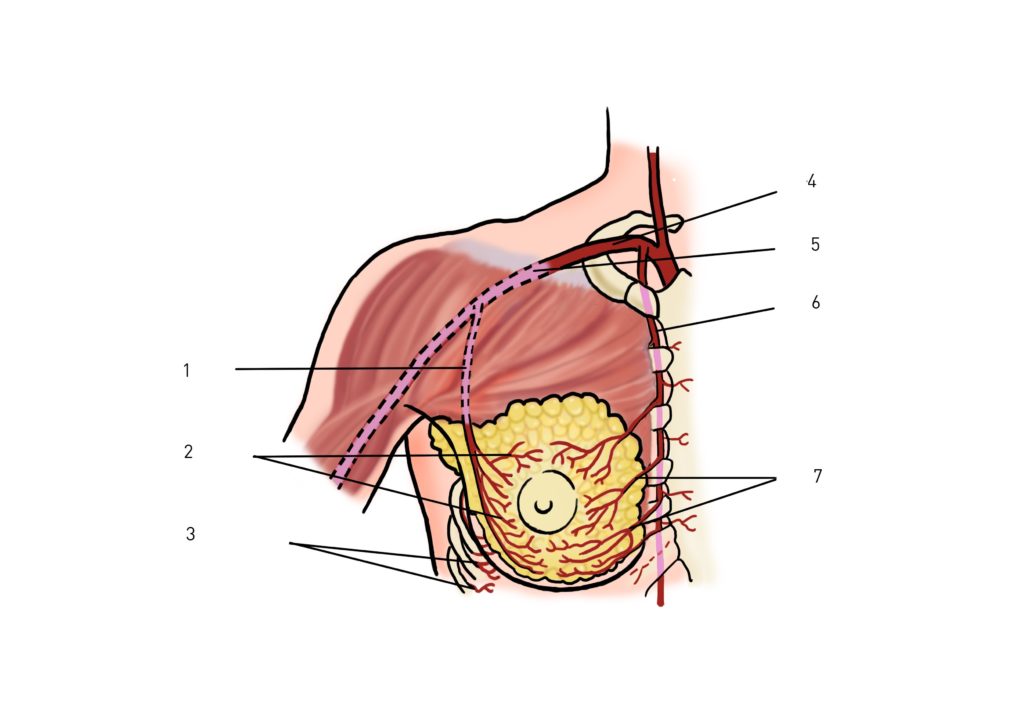


*Figure 3*

1. Lateral thoracic artery
2. Lateral mammary arteries
3. Anterior intercostal arteries
4. Posterior mammary arteries
5. Posterior intercostal arteries
6. **The following localization techniques are used before the operation in non-palpabel tumors:**
7. Radio guided wire; radio guided seed
8. Radio guided wire; ultrasound imaging
9. Radio guided wire; blue dye injection
10. Radio guided seed; blue dye injection
11. Radio guided seed; ultrasound imaging
12. **What is the caudal border of the incision line in the axilla when performing an axillary lymph node dissection?**
13. 1-2 cm above the axillary hair line
14. 4-5 cm below the axillary hairline
15. At the level of the fifth intercostal space
16. 1-2 cm below the axillary hairline
17. At the level of the fourth intercostal space
18. **Which muscle forms the dorsal border of the axillary lymph node dissection?**
19. Latissimus dorsi
20. Subscapularis
21. Pectoralis minor
22. Serratus anterior
23. Pectoralis major
24. **Injury to which nerve can cause winging of the scapula?**
25. Axillary
26. Radial
27. Thoracodorsal
28. Intercostobrachial
29. Long thoracic
30. **Which structure is indicated by number 2 in *Figure 4*?**


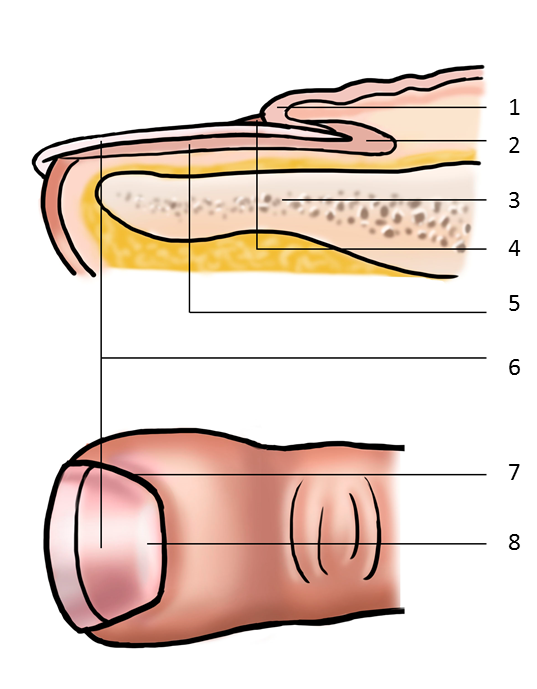


*Figure 4*

1. Nail bed
2. Proximal nail fold
3. Cutilate
4. Lateral nail fold
5. Nail matrix
6. **In which order is a toenail avulsion performed in the case of an ungus incarnates?**
7. Transection of the nail plate
8. Excision of the nail matrix
9. Vaseline application
10. Digital nerve block anesthesia
11. Disinfection

Answer: _________________________

1. **When should a lipoma be sent in for pathological examination?**
2. If it is located on the thigh
3. If the diagnosis is uncertain
4. Lipoma > 2 cm
5. If it is located on the trunk
6. If a lipoma recurs
7. **What is NOT a common location for a lipoma?**
8. Leg
9. Trunk
10. Arm
11. Thigh
12. Back of the neck
13. **Which structure should be opened to expose the peritoneum during the midline incision?**
14. Linea alba
15. Rectus muscle
16. External oblique muscle
17. Anterior rectus sheath
18. Posterior rectus sheath
19. **When performing a midline incision of the abdominal wall, which structure(s) should be avoided while incising the peritoneum?**
20. Intra-abdominal organs
21. Superior epigastric vessels
22. Superficial epigastric vessels
23. Preperitoneal fat
24. Rectus abdominis muscle

**Thank you for your participation!**

Department of Surgery, Erasmus University Medical Center
