# Supplementary material for: Standardized videos in addition to the surgical curriculum in Medical Education for surgical clerkships: a cohort study
Source: BMC Med Educ. 2022 May 19;22:384. doi: 10.1186/s12909-022-03314-w (PMC9121575; doi:10.1186/s12909-022-03314-w)
Supplement: Supplementary file 3 — Additional file 3. Student questionnaire - T1 - Demographics - control group. [file 12909_2022_3314_MOESM3_ESM.docx]

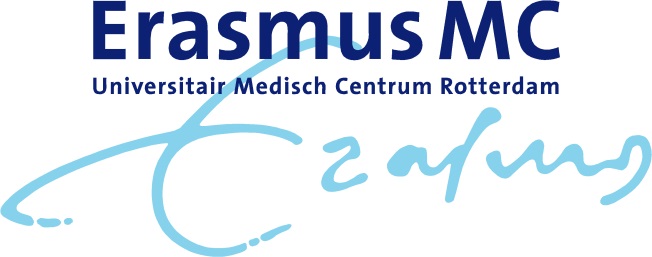


**EMC department of surgery: Surgical Internship and Multimedia**

This questionnaire is made by the department of Surgery to study the use of multimedia of interns during the clinical part of the internship.

Dear student,

The department of Education of Surgery in the Erasmus Medical Center is currently conducting a study on the use of multimedia (books, internet sources etc.) during the internship. The results will be used to improve the current curriculum. Therefore, we would kindly like to ask you to fill out the following forms (20 min. approximately). There will be 3 gift cards awarded via lottery (€20,- bol.com).

Please note that the information from these questionnaires will **not** be used in the final grading of the internship or and does not have any other implications. Your contact information will only be used to contact you if you won one of the gift cards.

By signing this form, I agree to the use of my information by the department of surgery.

| Name |  |
| --- | --- |
| Student number |  |
| E-mail |  |
| Date |  |
| Signature |  |

**Internship**

1. Practical (work/student job) experiences in hospital before current internship

- Skillslab (anatomy)
- Operating rooms (e.g. perfusion team)
- Nutrition team (surgical ward)
- Acute care student team
- Zorgacademie
- Education Service Center (onderwijs service centrum)
- No practical experience
- Experience in another non-surgical department
- Other: ……………………………………………..

1. Where did you do your surgery internship? *

- Admiraal de Ruyter ziekenhuis
- Albert Schweitzer ziekenhuis
- Amphia ziekenhuis
- Bravis ziekenhuis
- Elisabeth TweeSteden ziekenhuis
- Erasmus Medisch Centrum
- Ikazia ziekenhuis
- Ijsselland ziekenhuis
- Maasstad ziekenhuis
- Reinier de Graaf Gasthuis
- Sint Franciscus Gasthuis en Vlietland
- Van Weel Bethesda ziekenhuis

1. I would like to pursue a career in a surgical specialty


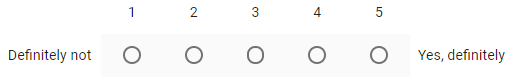


1. How many procedures did you attend (approximately) during this internship?

____________________________________________________________ (number)

1. During how many of the procedures that you attended (approximately) did you scrub in?

____________________________________________________________ (number)

1. Which of the following procedures did you attend during your internship?

*Multiple answers possible*

- Toenail avulsion
- Open Inguinal Hernia Repair
- Laparoscopic Cholecystectomy
- Laparoscopic Appendectomy
- Lumpectomy - Mamma
- Sentinel Node procedure
- Lipoma Excision
- Laparotomy - Abdominal Wall Incision
- Laparoscopic Right Colectomy
- Open Colectomy
- None of the above

1. I feel I have a good understanding of the essential procedures that I did NOT attend *


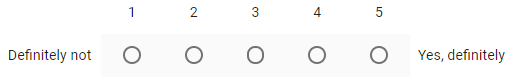


1. I feel I have a good understanding of the surgical procedures that I attended, other than the ten essential procedures


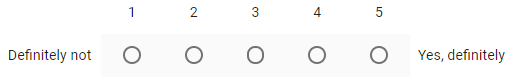


**Available sources**

Books, reliable internet sources etc.

1. I feel I have sufficient sources to prepare myself for the surgical internship(s)


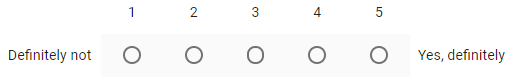


1. I feel I have sufficient sources to prepare myself for basic surgical skills


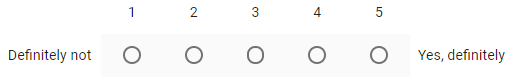


1. I feel I have sufficient sources to prepare myself for general procedures


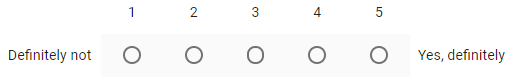


1. I feel I have sufficient sources to prepare myself for surgical procedures


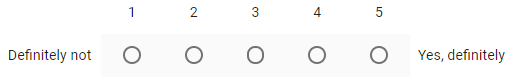


1. I feel I have sufficient sources to study surgical anatomy


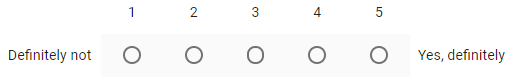


**Surgical knowledge**

1. I feel I have gained sufficient knowledge during my the surgical internship


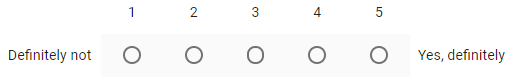


1. I feel I have sufficient knowledge about basic surgical skills

*For example: knots, sutures, instruments*


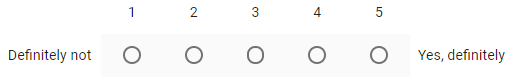


1. I feel I have sufficient knowledge of general procedures

*Catheter placement, I.V., etc.*


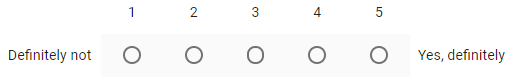


1. I feel I have sufficient knowledge about surgical procedures


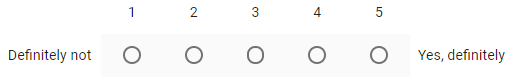


1. I feel I have sufficient knowledge about operations and surgical procedures in general


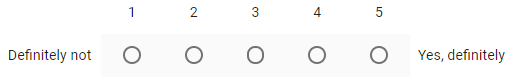


1. I feel I have sufficient knowledge about complications after surgery


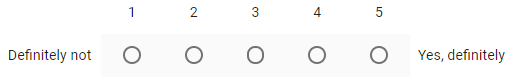


1. I feel I have sufficient knowledge about the objectives of surgical procedures


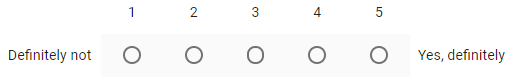


1. I feel I have sufficient knowledge about the do's and don'ts on the O.R.


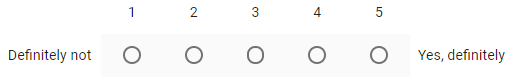


1. I feel I have sufficient surgical anatomical knowledge


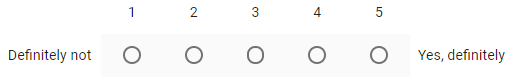


**Multimedia: Books, reliable internet sources etc.**

1. Which medium did you use most during this internship? *Please state one medium*

Books, internetsites, youtube channels etc. (Specify the name of the source. Reply in Dutch / English).

|  |
| --- |
|  |

2. What other medium/media did you use during this internship? *Multiple answers possible*

Books, internetsites, youtube channels etc. (Specify the name of the source. Reply in Dutch / English).

|  |
| --- |
|  |
|  |
|  |

3. Multimedia was helpful for my understanding of surgical procedures


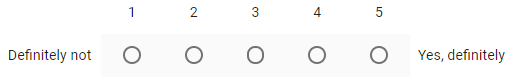


4. Multimedia was helpful for my understanding of basic surgical skills


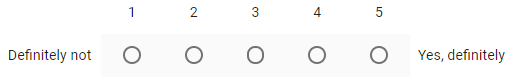


1. Multimedia was helpful for my understanding of surgical anatomy


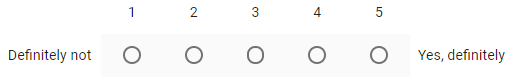


1. How much time did you spend on average on the preparation of a procedure?

___________ (minutes per procedure on average)

1. The content of the multimedia I used is of high quality


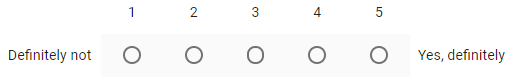


1. The multimedia I used was user friendly


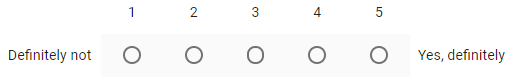


1. I can apply the content of multimedia in the future during my studies or work


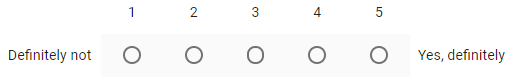


10. I can apply the content of multimedia in the future during my studies

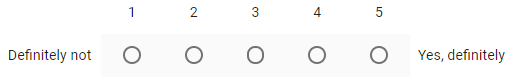


11. I have the impression that my knowledge has expanded on a long-term basis by using multimedia


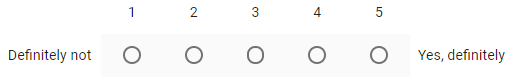


12. I miss a medium to optimally prepare myself during my surgical internship


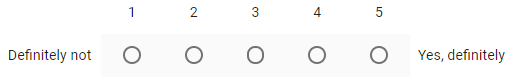


13. If so, what would your ideal medium look like?

|  |
| --- |
|  |
|  |
|  |
