# Supplementary material for: Standardized videos in addition to the surgical curriculum in Medical Education for surgical clerkships: a cohort study
Source: BMC Med Educ. 2022 May 19;22:384. doi: 10.1186/s12909-022-03314-w (PMC9121575; doi:10.1186/s12909-022-03314-w)
Supplement: Supplementary file 5 — Additional file 5. Student questionnaire - T1 - Surgical knowledge. [file 12909_2022_3314_MOESM5_ESM.docx]

**Appendix 5 – Surgical knowledge – T1**

1. **The critical view of safety should be obtained during the cholecystectomy. Which duct should be isolated?**
2. Common bile duct


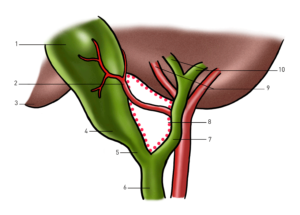


*Figure 1*

1. Left hepatic duct
2. Right hepatic duct
3. Common hepatic duct
4. Cystic duct
5. **Which structure is located inside the cystohepatic triangle in *Figure 1*?**
6. Common hepatic artery
7. Cystic artery
8. Left hepatic artery
9. Cystic duct
10. Common hepatic duct
11. **Which complication may arise due to injury to the iliohypogastric nerve?**
12. Loss of sensation of the inguinal and suprapubic region
13. Loss of sensation over the outer thigh
14. Weakness of the iliacus muscle
15. Weakness of the psoas muscle
16. Loss of sensation over the inner thigh
17. **Which vessels are at risk of injury during the skin incision and subcutaneous tissue incision?**
18. Inferior epigastric


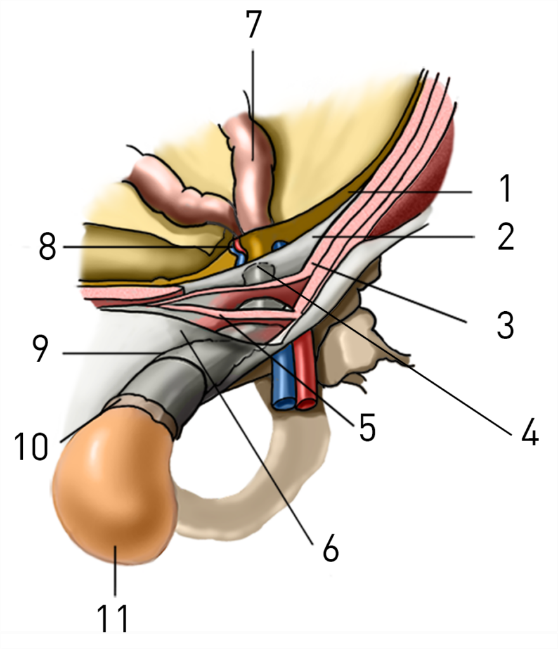


*Figure 2*

1. Superficial epigastric
2. Cremasteric
3. Femoral
4. Superior epigastric
5. **Which number points out the internal oblique muscle in Figure 2?**
6. 5
7. 6
8. 10
9. 3
10. 2
11. **In a right colectomy the bowel is resected between the which of the following structures?**
12. caecum; splenic flexure
13. caecum; proximal transverse colon
14. caecum; hepatic flexure
15. terminal ileum; hepatic flexure
16. terminal ileum; proximal transverse colon
17. **Which of the following complications are known to occur following a right colectomy?**

*Multiple answers possible*

1. Ureteral injury
2. Postoperative ileus
3. Decreased renal function


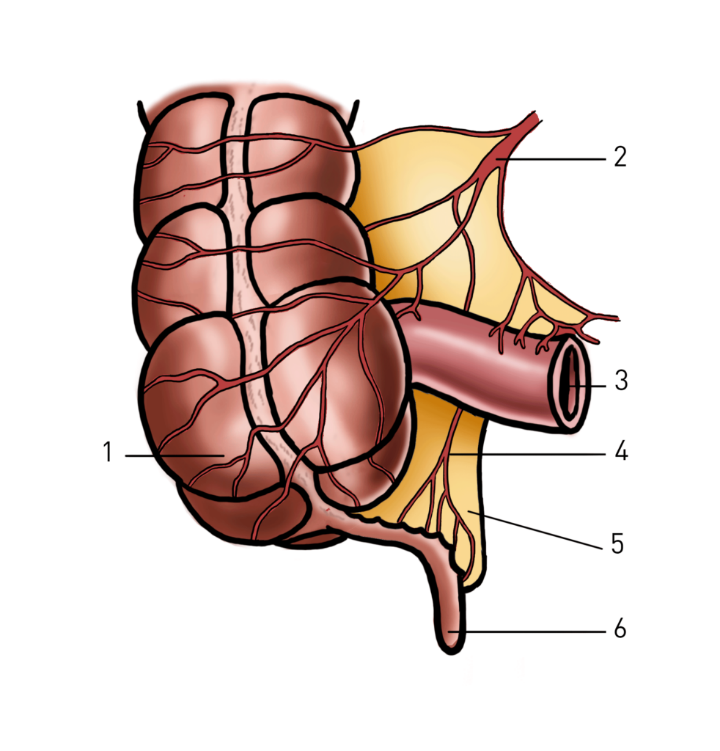


*Figure 3*

1. Anastomotic leakage
2. **What is the most common origin of the right colic artery?**
3. Directly from the superior mesenteric artery
4. Branch of the middle colic artery
5. Common trunk with the ileocolic artery
6. Branch of the ileocolic artery
7. Common trunk with the middle colic artery
8. **What structure is indicated by number 5 in *Figure 3***?
9. Lymphatic vessels
10. Appendix window
11. Peritoneum
12. Meso-appendix
13. Sigmoid mesentery
14. **In** **order to gain vascular control during appendectomy, the ... artery, located in the ... is transected at the ... of the appendix.**

A. ileocecal; mesocolon; base

B. appendicular; mesoappendix; base

C. appendicular; mesocolon; tip D. ileocecal; mesoappendix; tip

E. appendicular; mesoappendix; tip

1. **After excision of large lipomas, the lipoma dead space can be closed using subcutaneous interrupted sutures. What is prevented by using this technique?**
2. Irritation of the skin
3. Recurrence of the lipoma
4. Spreading to other body parts
5. Keloid formation
6. Seroma formation
7. **After de resection of a lipoma the risk of … will increase with ….**
8. infection; the formation of a hematoma
9. hematoma formation; the onset of an infection
10. seroma formation; the formation of a hematoma
11. recurrence; the onset of an infection
12. recurrence; the formation of a hematoma
13. **What are the two objectives of a lipoma excision?**
14. Spreading to other body parts and prevent infection
15. Eradicate pain and restore cosmetic appearance


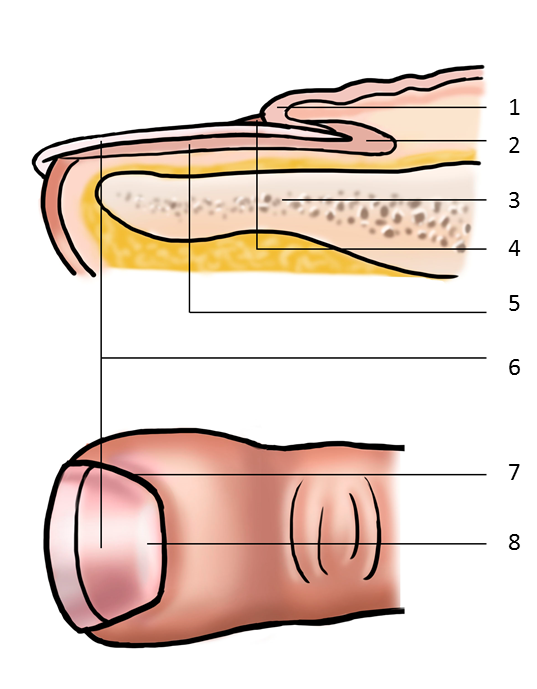


*Figure 4*

1. Prevent infection and eradicate pain
2. Spreading to other body parts and restore cosmetic appearance
3. Spreading to other body parts and eradicate pain
4. **Which structure is indicated by number 5 in *Figure 4*?**
5. Nail bed
6. Proximal nail fold
7. Cutilate
8. Lateral nail fold
9. Nail matrix
10. **Incomplete removal of nail segments in the case of an unguis incarnates could lead to…**

*Multiple answer may be correct.*

1. Recurrence
2. Chronic infection
3. A retained nail fragment
4. **Which therapy is the most suitable curative alternative for resectable breast cancer, if a lumpectomy is contraindicated?**
5. Radical mastectomy
6. Radiotherapy
7. Systemic therapy
8. Hormone therapy
9. Radiochemotherapy
10. **What is caused when the long thoracic nerve is injured?**

A. Paralysis of the latissimus dorsi muscle causing winging of the clavicula.

B. Numbness at the lateral side of the thorax.

C. Pain at the lateral side of the thorax.

D. Paralysis of the serratus anterior muscle causing winging of the scapula. E. Paralysis of the latissimus dorsi muscle causing winging of the scapula.


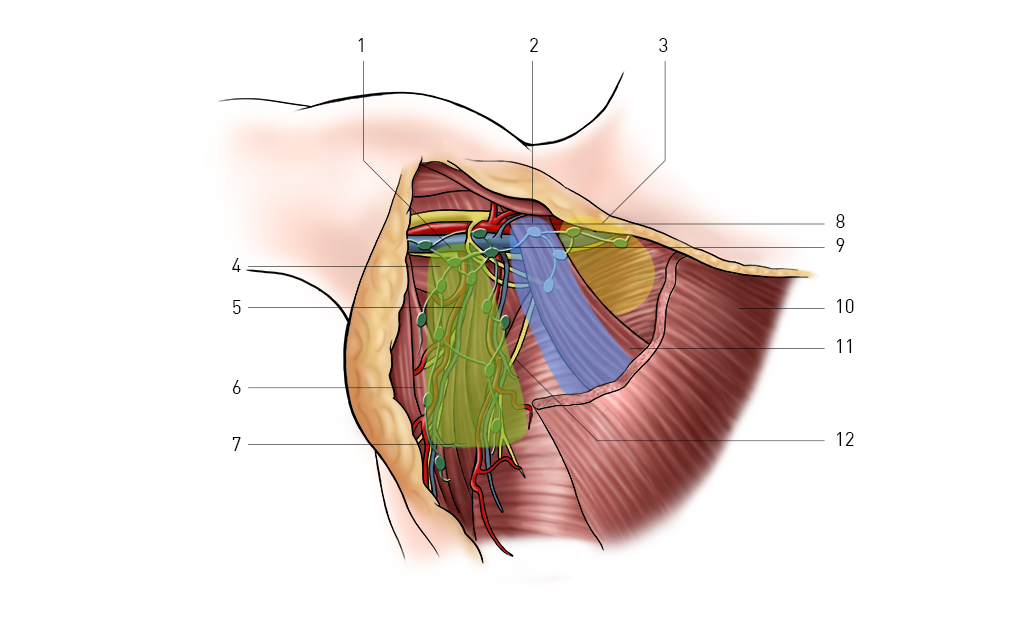


*Figure 5*

1. **Which muscle is pointed out by number 11 in *Figure 5*?**
2. Latissimus dorsi
3. Pectoralis minor
4. Pectoralis major
5. Subscapularis
6. Serratus anterior
7. **What could result if the facial sutures are pulled too tightly after a midline incision of the abdominal wall (laparotomy)?**
8. Necrosis


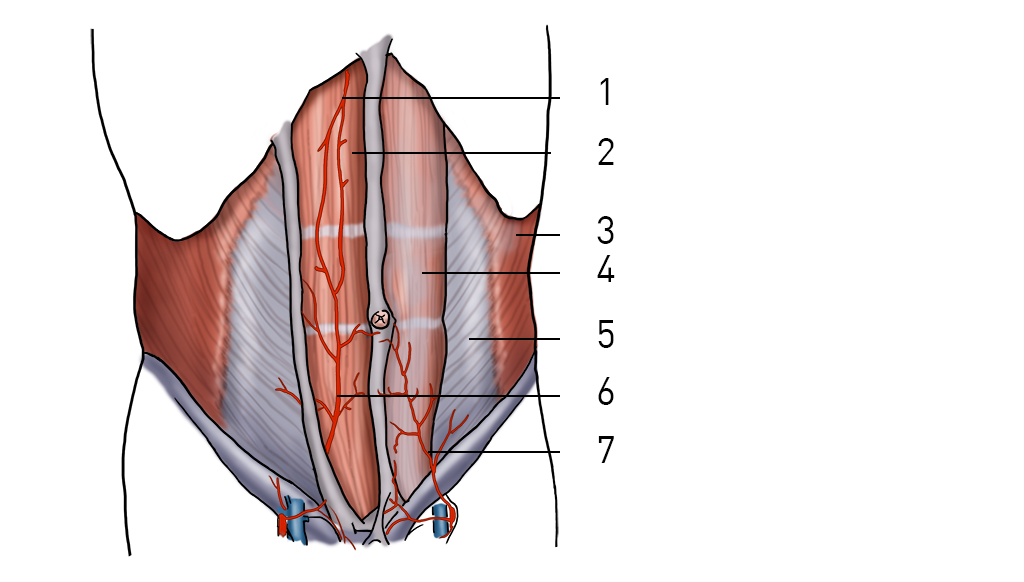


*Figure 6*

1. Infection
2. Seroma formation
3. Hemorrhage
4. Dehiscence
5. **Which artery is indicated by number 6 in *Figure 6*?**
6. Inferior epigastric
7. Femoral
8. Superficial circumflex iliac
9. Superior epigastric
10. Superficial epigastric

**Thank you for you participation!**

Department of Surgery, Erasmus Univeristy Medical Center
