# Supplementary material for: Standardized videos in addition to the surgical curriculum in Medical Education for surgical clerkships: a cohort study
Source: BMC Med Educ. 2022 May 19;22:384. doi: 10.1186/s12909-022-03314-w (PMC9121575; doi:10.1186/s12909-022-03314-w)
Supplement: Supplementary file 6 — Additional file 6. Supervisor questionnaire - T1. [file 12909_2022_3314_MOESM6_ESM.docx]

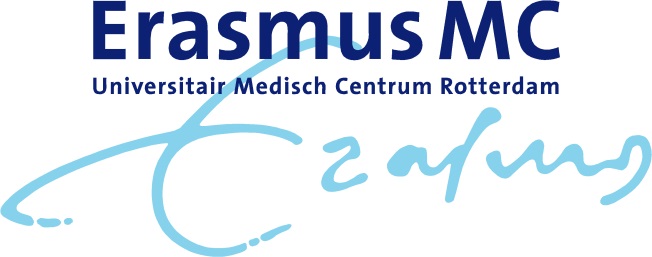


**Supervisors**

1. How long have you been a supervisor for interns?

_______________ (years)

1. Where do you work?

- Admiraal de Ruyter ziekenhuis
- Albert Schweitzer ziekenhuis
- Amphia ziekenhuis
- Bravis ziekenhuis
- Elisabeth TweeSteden ziekenhuis
- Erasmus Medisch Centrum
- Ikazia ziekenhuis
- Ijsselland ziekenhuis
- Maasstad ziekenhuis
- Reinier de Graaf Gasthuis
- Sint Franciscus Gasthuis en Vlietland
- Van Weel Bethesda ziekenhuis

**Interns**

1. How many interns were working in your hospital during the study period?

_______________ (number)

1. How many of the above-mentioned number of interns did you observe yourself during this period?

_______________ (number)

1. Have you received input from your colleagues about the knowledge and behavior of the other interns during this period?

- Yes
- No

1. The interns have sufficient knowledge about surgical procedures


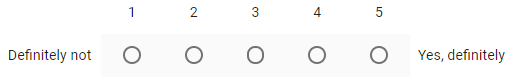


1. The interns have sufficient knowledge about objectives of surgical procedures


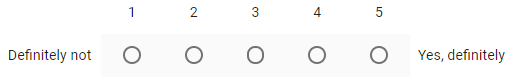


1. The interns have sufficient knowledge about complications after surgery


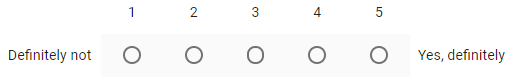


1. The interns have sufficient knowledge about surgical anatomy


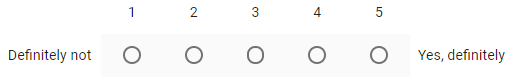


1. The interns have sufficient knowledge about basic surgical skills


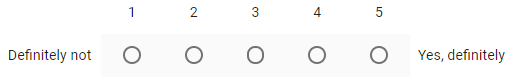


1. The interns have sufficient knowledge about the do's and don'ts on the O.R


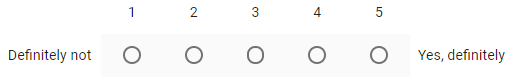


**Available multimedia sources**

1. I have the impression that interns have sufficient sources to prepare themselfs for surgical procedures


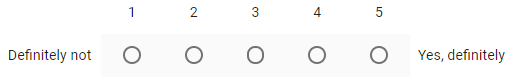


1. I have the impression that interns have sufficient sources to prepare themselfs for basic surgical skills


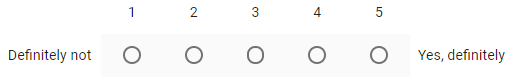


1. I have the impression that interns have sufficient sources to study the surgical anatomy


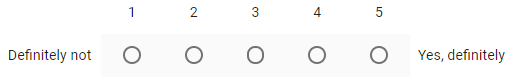


1. I have the impression that interns use the following sources to prepare themselfs for the internship


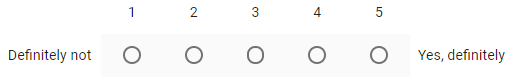


1. I have the impression that interns are lacking high quality sources to prepare themselfs for the internship


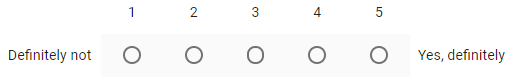


1. If so, please describe the extra sources students should be provided

|  |
| --- |
|  |
|  |
|  |

**Intern confidence**

1. I would rate the self-confidence of interns during their internship in general as

*In the surgical ward, emergency department, outpatient clinic etc.*


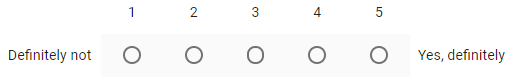


Very confident

Very insecure

1. I would rate the self-confidence of interns in the O.R. as


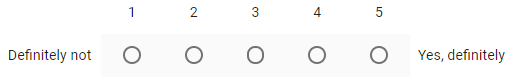


Very confident

Very insecure

**Thank you for your participation!**
